# Application of robust regression in translational neuroscience studies with non-Gaussian outcome data

**DOI:** 10.3389/fnagi.2023.1299451

**Published:** 2024-01-24

**Authors:** Michael Malek-Ahmadi, Stephen D. Ginsberg, Melissa J. Alldred, Scott E. Counts, Milos D. Ikonomovic, Eric E. Abrahamson, Sylvia E. Perez, Elliott J. Mufson

**Affiliations:** ^1^Banner Alzheimer’s Institute, Phoenix, AZ, United States; ^2^Department of Biomedical Informatics, University of Arizona College of Medicine-Phoenix, Phoenix, AZ, United States; ^3^Center for Dementia Research, Nathan Kline Institute, Orangeburg, NY, United States; ^4^Department of Psychiatry, New York University Grossman School of Medicine, New York, NY, United States; ^5^Department of Neuroscience and Physiology, New York University Grossman School of Medicine, New York, NY, United States; ^6^NYU Neuroscience Institute, New York University Grossman School of Medicine, New York, NY, United States; ^7^Departments of Translational Neuroscience and Family Medicine, Michigan State University, Grand Rapids, MI, United States; ^8^Department of Neurology, University of Pittsburgh School of Medicine, Pittsburgh, PA, United States; ^9^Geriatric Research Education and Clinical Center, VA Pittsburgh Healthcare System, Pittsburgh, PA, United States; ^10^Department of Psychiatry, University of Pittsburgh, Pittsburgh, PA, United States; ^11^Department of Translational Neurosciences, Barrow Neurological Institute, Phoenix, AZ, United States

**Keywords:** robust regression, linear regression, normal distribution, Gaussian distribution, normality assumption, Alzheimer’s disease

## Abstract

Linear regression is one of the most used statistical techniques in neuroscience, including the study of the neuropathology of Alzheimer’s disease (AD) dementia. However, the practical utility of this approach is often limited because dependent variables are often highly skewed and fail to meet the assumption of normality. Applying linear regression analyses to highly skewed datasets can generate imprecise results, which lead to erroneous estimates derived from statistical models. Furthermore, the presence of outliers can introduce unwanted bias, which affect estimates derived from linear regression models. Although a variety of data transformations can be utilized to mitigate these problems, these approaches are also associated with various caveats. By contrast, a robust regression approach does not impose distributional assumptions on data allowing for results to be interpreted in a similar manner to that derived using a linear regression analysis. Here, we demonstrate the utility of applying robust regression to the analysis of data derived from studies of human brain neurodegeneration where the error distribution of a dependent variable does not meet the assumption of normality. We show that the application of a robust regression approach to two independent published human clinical neuropathologic data sets provides reliable estimates of associations. We also demonstrate that results from a linear regression analysis can be biased if the dependent variable is significantly skewed, further indicating robust regression as a suitable alternate approach.

## Introduction

### Linear regression and the assumption of normality

Linear regression analysis is among the most used statistical approaches to examine associations between continuous variables including the field of neuroscience. This statistical approach is a standard function available in statistical software packages with outputs that are interpreted in terms of per-unit increases or decreases making data interpretation accessible to investigators with varying levels of statistical training and expertise.

Despite the simplicity and accessibility of linear regression analyses, an important, but often ignored, assumption is that the dependent variable error follows a Gaussian, or normal, distribution (Lang, 2004; Strasak et al., 2007). Although some degree of skewness in the distribution of a dependent variable can be tolerated without invalidating the results of a given model (Ghasemi and Zahediasl, 2012), linear regression models generated from data with highly skewed or bimodal distributions likely yield spurious or invalid results (Hoekstra et al., 2012). This problem is amplified in studies with relatively small sample sizes where the magnitude of associations may be unduly increased or decreased due to the degree of skewness in the dependent variable (Hoekstra et al., 2012).

### Determining whether a variable meets the assumption of normality

Several established visual and quantitative approaches are used to assess whether a variable meets the assumption of normality (Ghasemi and Zahediasl, 2012). Although the most common qualitative approach is to create a histogram of the dependent variable and determine whether the shape of the histogram is consistent with a Gaussian distribution, quantitative approaches to test the assumption of normality are also useful. The Shapiro–Wilk test (Shapiro and Wilk, 1965) uses statistical significance to indicate whether a variable’s error profile follows a Gaussian distribution. For the Shapiro–Wilk test, value of ps that are ≤0.05 indicate that the variable’s error *is not* consistent with a normal distribution and may not be amenable to the use of parametric statistics (e.g., linear regression). Since the Shapiro–Wilk test is sensitive to the highest and lowest values in large datasets (*n* > 500), it may erroneously indicate that a data set does not meet the assumption of normality even though visual inspection indicates otherwise (Uttley, 2019). Parametric tests, including linear regression, are fairly robust to deviations from normality in large sample sizes (Schmider et al., 2010) which allows for results to remain valid despite some degree of skewness in the datasets (Schmider et al., 2010; Ghasemi and Zahediasl, 2012; Rochon et al., 2012). Despite the availability of visual and statistical tools to examine the normality of a variable, the question remains how to analyze data that do not meet the assumption of normality, particularly with smaller sample sizes.

### Logarithmic transformation of data

**Figure 1:**
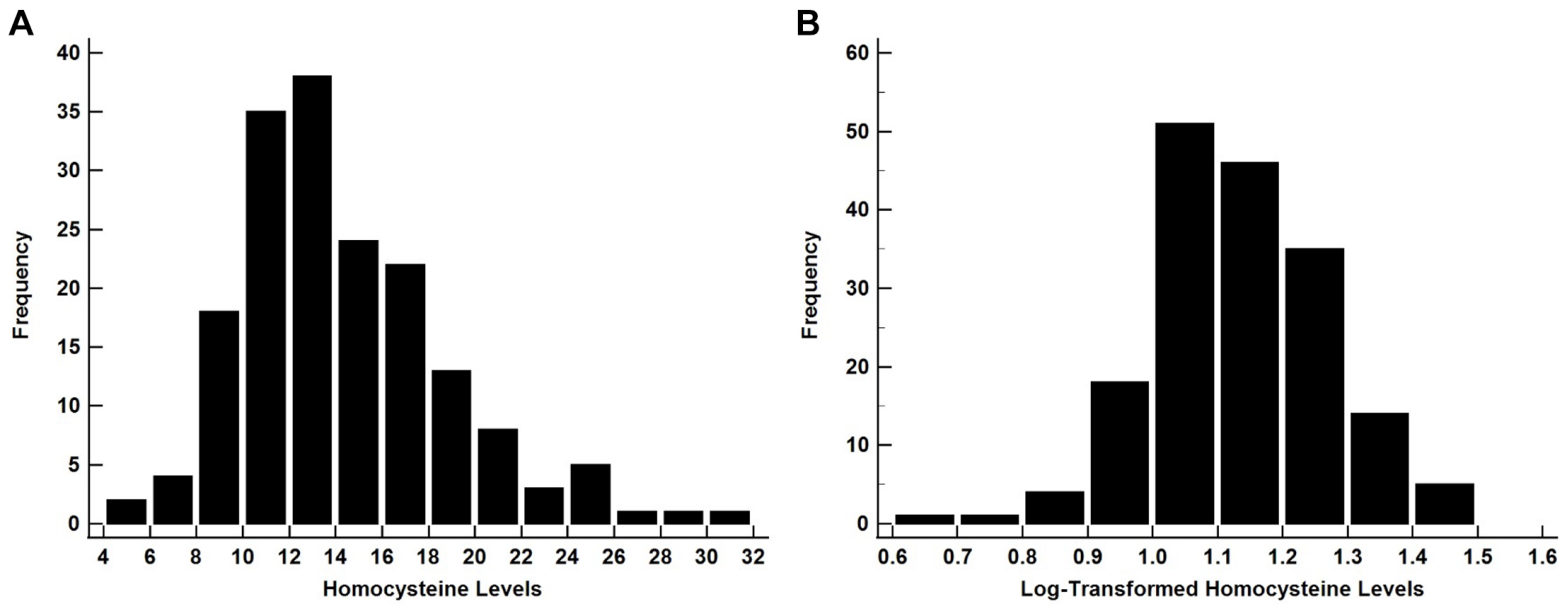
The histograms showing the distribution of raw **A** and **B** log-transformed Homocysteine values. Since the raw data are right-skewed, the log-transformed data yielded a Gaussian-like distribution allowing for the use of for parametric test. Homocysteine data are from Malek-Ahmadi et al. (2013).

A common approach to handling skewed data is to apply a logarithmic (log) transformation of values that will result in the data meeting the assumption of normality (Feng et al., 2013). Since log-transformations have the effect of moving the center of the distribution from left to right (Feng et al., 2013), this method should only be used when the data are right-skewed. Data shown in Figures 1A,B illustrate how the application of a log-transformation on right skewed data shifts the shape of the distribution so that it is closer to a Gaussian distribution.

The application of Shapiro–Wilk tests on each data set confirmed that the raw data do not meet the assumption of normality (Figure 1A, *P* < 0.001), while log-transformation of the data supports normality (Figure 1B, *P* = 0.20). Conversely, for left-skewed distributions log-transformation only exacerbates the skewness of the variable (Feng et al., 2013). There are also instances where data are so heavily right-skewed that log-transformed values will not meet the assumption of normality (Ravaglia et al., 2006).

Another limitation of log transformations concerns data scaling and interpretation. Since log transformation is a form of scaling, the variable’s original unit of measure is no longer used following transformation (Bland and Altman, 1996). In studies involving a clinical or practical interpretation, reporting a dependent variable on the log scale has limited utility. For example, a total cholesterol value of 189 mg/dL is easily interpreted and has clinical and pathological meaning. However, a log-transformed total cholesterol value of 2.2764 would not be useful for a practical interpretation.

### Dichotomizing continuous variables

A common, yet methodologically unsound practice is to dichotomize a skewed continuous variable at a particular value in its distribution. This approach is not favored due to the loss in statistical power that results from collapsing a continuous variable into two categories (Kuss, 2013). Another caveat of dichotomization is that the selected cut point is often the mean, median, or some other arbitrary value. If the selection of a cut point does not have a scientific or clinical rationale, the result is difficult to interpret and limits its translation to another dataset containing the same variable. In cases where there is an established cut point for a continuous variable (e.g., hemoglobin A1c > 5.7; Mini Mental State Exam <26), dichotomization may be acceptable (Ragland, 1992), but the preferred analytic approach is to maintain the variable’s continuous scale.

### Robust regression as an alternative to linear regression

While parametric tests like the t-test, analysis of variance (ANOVA), and Pearson correlation have non-parametric counterparts (e.g., Mann–Whitney, Kruskall-Wallis and Spearman correlation, respectively), a non-parametric counterpart to linear regression is lacking. Generalized linear models (GLMs) specify an underlying error distribution for a dependent variable used in estimating the regression model (Neuhaus and McCulloch, 2011). However, there are limits to the kinds of distributions that can be specified (Neuhaus and McCulloch, 2011). In addition, this model may be an impediment to a large number of investigators that lack training in advanced applied statistics.

Robust regression analysis should be used more widely in situations where a dependent variable’s error distribution does not lend itself to well-known parametric statisitcs. Although robust regression methodology has existed for several years (Wainer and Thissen, 1976; Hettmansperger et al., 2000; Cantoni and Ronchetti, 2006; Maronna et al., 2006) and its interpretation is similar to linear regression, it is not typically part of graduate-level statistics and methodology courses taught in neuroscience programs.

The primary difference between linear regression and robust regression is that the former regresses individual datapoints using the mean of the dependent variable, while robust regression uses Maximum likelihood (M)-estimators as the regressor (Huber, 1964; Maronna and Yohai, 2000; Valdora and Yohai, 2014; Varin and Panagiotakos, 2019; Yang et al., 2019). A strength of M-estimators in robust regression is that it allows for valid associations to be drawn in the presence of outliers and significant skewness in a continuous dependent variable (Cantoni and Ronchetti, 2006).

Here, we show how robust regression can be used in datasets where a dependent variable’s error distribution does not meet the assumption of normality. Using two independent datasets from postmortem human brain tissue assays, we demonstrate how the use of linear regression with a skewed dependent variable yields biased estimates of associations. By contrast, we discuss how robust regression enables reliable estimates when dependent variables are skewed.

## Methods

### Data sources

Dataset 1 comes from a study that investigated neurotrophin receptor expression via single population microarray analysis within the hippocampal CA1 sector (Ginsberg et al., 2019). These data were obtained in postmortem tissue samples from particpants of the Rush Religious Orders Study (RROS) that came to autopsy with an antemortem clinical diagnoses of no cognitive impairment (NCI, *n* = 13), mild cognitive impairment (MCI, *n* = 15), and Alzheimer’s disease (AD, *n* = 9; Ginsberg et al., 2019). Exclusion criteria included no other neurological diagnoses (e.g., Parkinson’s disease, Lewy body disease, hippocampal sclerosis or large cerebral infarcts). Participants were not taking cholinesterase inhibitors. Postmortem neuropathological evaluation, demographics and APOE genotype was available for each group. For the purpose of the present study, we analyzed data obtained for the BDNF TrkB and neurotrophin-3 receptors TrkC receptors.

Dataset 2 was derived from a study investigating the association between vesicular glutamate transporters and spinophilin with last ante-mortem clinical and postmortem neuropathological diagnoses as well as quantitative cyano-PiB- and X-34-stained amyloid plaque loads in the precuneus (Mi et al., 2023). Antemortem clinical, demographic, APOE information and exclusion criteria were same as for Dataset 1. The dataset was comprised of NCI (*n* = 19), MCI (*n* = 10), and mild AD (*n* = 7) cases from the RROS cohort and end stage AD (*n* = 10) cases from the University of Pittsburgh Alzheimer’s Disease Research Center.

### Robust regression

The robust regression approach used for these analyses utilized the M-estimation approach (Huber, 1964) where the residual function of the regression model is minimized as opposed to the sum of squared errors which is used in typical linear regression models (Yohai, 1988; Yohai, 1991; Abonazel and Kamel, 2019; Awwad et al., 2022). This allows for the regression estimates to be more resistant to the influence of outliers and allows their use when there is no scientific or methodologic reason to exclude the outliers (Abonazel and Kamel, 2019; Awwad et al., 2022).

### Statistical analysis

For the analysis of Dataset 1, TrkB expression was a dependent variable with a global cognitive score (GCS) comprised of a battery of 19 cognitive tests (Ginsberg et al., 2019) as the independent variable in both linear and robust regression models. The second example used TrkC expression as the independent variable and entorhinal cortex neurofibrillary tangle (NFT) counts as the dependent variable (Ginsberg et al., 2019). The Shapiro–Wilk test was used to determine whether each of the dependent variables met the assumption of normality. Visual inspection of the dependent variables’ distributions was presented as histograms. Dataset 1 regression models included age at death, sex, years of education, and APOE ε4 carrier status as covariates. Spearman correlations were also used to assess the possible influence of multicollinearity among the predictor variables in each model.

For Dataset 2, cyano-PiB- and X-34-stained amyloid plaque loads in precuneus were used as dependent variables with MMSE score as the independent variable (Mi et al., 2023). The Shapiro–Wilk test was used to determine whether each of the dependent variables met the assumption of normality. Visual inspection of the dependent variables’ distributions was also shown using histograms. Dataset 2 regression models included age at death, sex, and years of education as covariates.

For all regression models, the coefficients, standard errors of the coefficients, and respective value of ps were compared qualitatively between the linear and robust models to show how these parameters are impacted by the choice of regression model. Multiple r-squared (*R*^2^) values were also reported for each model as a measure of model fit. Although adjusted R^2^ values are the preferred method for assessing model fit when a regression model has multiple independent variables, adjusted R^2^ is not available for robust regression. Therefore, for consistency we used the simple multiple *R*^2^ for each model.

Permutation test linear regression models served as an additional reference for the robust regression models. Permutation tests lack any assumptions about the error distribution of a dataset and estimates are generated by re-sampling the raw data over many iterations to derive the *p*-values (Mangiafico, 2016). The Exact permutation method was used to test all possible permutations of the dependent variable.

Statistical analyses were carried out using the ‘robust’, ‘robustbase’, and ‘lmPerm’ packages in R 4.1.3 (R Core Team, 2022).

## Results

### Dataset 1

Demographic, cognitive, and neuropathologic data for the NCI, MCI, and AD cases are shown in Table 1.

**Table 1:** Demographic, clinical, and neuropathologic characteristics of rush religious orders study cases used in Dataset 1 (A) and Dataset 2 (B).

A			
	NCI (*n* = 13)	MCI (*n* = 15)	AD (*n* = 9)
Age at death (years)	82.95 ± 7.70	85.29 ± 4.51	86.84 ± 6.55
Education (years)	17.46 ± 4.07	19.13 ± 2.17	17.56 ± 1.67
Sex (M/F)	7/6	6/9	2/7
APOE ε4 carrier status (+/−)	1/12	7/8	8/1
MMSE	27.85 ± 1.57	26.80 ± 2.73	20.22 ± 4.06
Global cognitive score (z-score)	0.02 ± 0.27	−0.43 ± 0.25	−1.59 ± 0.37
Post-mortem interval (hours)	7.45 ± 8.19	6.92 ± 4.01	7.57 ± 3.57
Brain weight at autopsy (grams)	1,245.77 ± 170.39	1,239.73 ± 212.03	1,123.75 ± 152.59
CERAD No AD Possible AD Probable AD Definite AD	7 2 2 2	1 2 5 7	0 0 3 6
Braak Stage 0-II III-IV V-VI	5 8 0	2 8 5	1 2 6

B			
	NCI (*n* = 19)	MCI (*n* = 10)	AD (*n* = 17)
Age at death (years)	85.84 ± 5.47	87.27 ± 5.35	83.86 ± 7.72
Education (years)	17.71 ± 2.87	17.60 ± 2.17	16.35 ± 3.24
Sex (M/F)	7/12	4/6	8/9
APOE ε4 carrier status (+/−)	3/16	4/5*	8/8*
MMSE	28.53 ± 1.58	26.10 ± 3.51	16.35 ± 3.24
Global cognitive score (z-score)	−0.04 ± 0.29	−0.54 ± 0.36	−1.46 ± 0.40
Post-mortem interval (hours)	6.06 ± 2.41	6.44 ± 3.00	7.09 ± 3.51
Brain weight at autopsy (grams)	1,209.84 ± 140.50	1,186.70 ± 96.41	1,190.24 ± 95.75
CERAD No AD Possible AD Probable AD Definite AD	4 4 10 1	2 0 6 2	0 0 4 13
Braak Stage 0-II III-IV V-VI	8 10 1	1 7 2	0 6 11

NCI, no cognitive impairment; MCI, mild cognitive impairment; AD, Alzheimer’s disease; mean ± standard deviation; MCI and AD groups each had one case where APOE genotype data was not available.

**Figure 2:**
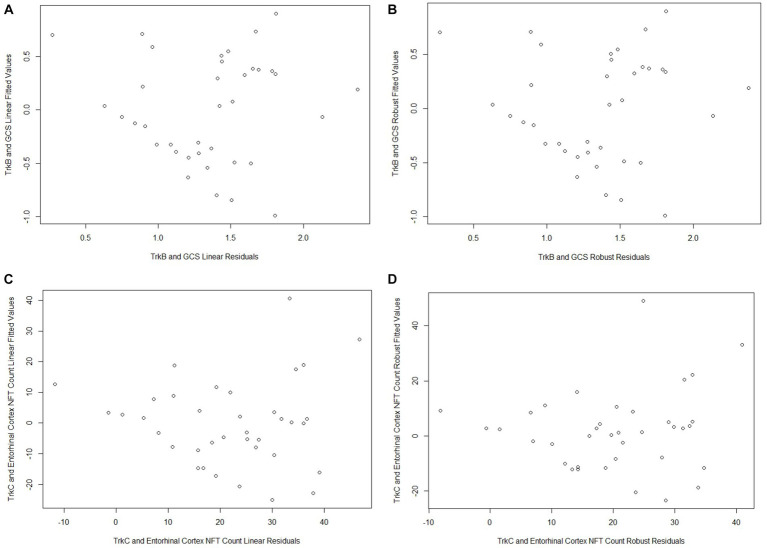
All of the plots indicate a lack of correlation between the fitted values and residuals from each model (**A** - Cyano-PiB and MMSE Linear Model; **B** - Cyano-PiB and MMSE Robust Model; **C** - X-34 and MMSE Linear Model; **D** - X-34 and MMSE Robust Model).

Both TrkB expression and entorhinal cortex NFT load did not meet the assumption of normality (*p* = 0.002 and *p* < 0.001, respectively; Table 2). GCS did not correlate with age (*r* = −0.27, *p* = 0.10) or education (*r* = 0.21, *p* = 0.21) and did not differ between males and females (*p* = 0.20). The linear regression model for GCS as a predictor of TrkB expression yielded a statistically significant association (*β* = 0.33, SE = 0.16, *p* = 0.04, *R*^2^ = 0.42) while the robust model indicated that this association was not statistically significant (*β* = 0.34, SE = 0.35, *p* = 0.34, *R*^2^ = 0.40; Table 3). While the two models produced similar regression coefficients and R^2^ values, the SE for the robust model was more than twice that of the linear model. In addition, the p-values of the associations in the two models diverged substantially and led to two very different interpretations. These differing results demonstrate that findings from the linear regression models are likely biased by the highly skewed distribution of TrkB expression values. In particular, the larger SE value in the robust regression model indicates that it is capturing more of the variability associated with the regression coefficient compared to the linear model. Residual plots for the linear and robust regression models are shown in Figures 2A,B revealed a lack of correlation between the fitted and residual values. For the permutation linear regression models, the regression coefficients were the same as those in the regular linear regression models. However, the GCS and TrkB association was no longer statistically significant (*p* = 0.06; Table 4) while the *R*^2^ value was nine percentage points lower. For the GCS and TrkB permutation model the results were achieved after five permutations while the TrkC and entorhinal cortex NFT permutation model required six permutations.

**Table 2:** Summary data for dependent variables used in the linear and robust regression models.

		NCI	MCI	AD	Shapiro–Wilk *p*-value*
Dataset 1					
	Trk2 Expression	1.93 ± 0.55	1.12 ± 0.54	0.92 ± 0.30	0.002
		2.06 (1.56, 2.21)	0.87 (0.69, 1.67)	0.76 (0.71, 1.03)	
	Entorhinal Cortex NFT Count	10.62 ± 9.99	24.13 ± 19.13	35.00 ± 20.43	<0.001
		5.00 (2.75, 20.00)	20.00 (12.75, 31.75)	34.00 (22.75, 42.25)	
Dataset 2					
	Cyano-PiB Load	1.47 ± 2.12	1.16 ± 1.00	6.11 ± 5.73	<0.001
		0.12 (0.00, 3.02)	0.88 (0.23, 1.99)	3.93 (1.89, 8.47)	
	X-34 Load	1.84 ± 2.47	2.24 ± 1.64	7.83 ± 7.21	<0.001
		0.45 (0.00, 4.01)	2.13 (1.74, 2.67)	5.60 (1.96, 12.13)	

NCI, no cognitive impairment; MCI, mild cognitive impairment; AD, Alzheimer’s disease; mean ± standard deviation; median (interquartile range); *p-values that are <0.05 indicate the data do not meet the assumption of normality.

**Table 3:** Comparison of regression statistics for linear and robust regression models.

		Linear regression results				Robust regression results			
		Coefficient	SE	*P*-value	*R* ^2^	Coefficient	SE	*P*-value	*R*^2^
Dataset 1									
	Global cognitive score and Trk2 expression	0.33	0.16	0.04	0.42	0.34	0.35	0.34	0.40
	Trk3 expression and entorhinal cortex NFT load	−15.18	6.50	0.03	0.46	−12.13	6.10	0.06	0.32
Dataset 2									
	MMSE and cyano-PiB load	−0.32	0.06	<0.001	0.54	−0.18	0.05	<0.001	0.21
	MMSE and X-34 load	−0.37	0.07	<0.001	0.52	−0.24	0.05	<0.001	0.15

**Table 4:** Results for permutation linear regression models.

		Coefficient	*P*-value	*R*^2^
Dataset 1				
	Global cognitive score and Trk2 expression	0.33	0.06	0.33
	Trk3 expression and entorhinal cortex NFT load	−15.18	0.03	0.37
Dataset 2				
	MMSE and cyano-PiB load	−0.32	<0.001	0.49
	MMSE and X-34 load	−0.37	<0.001	0.47

Permutation linear regression models do not produce SE values.

**Figure 3:**
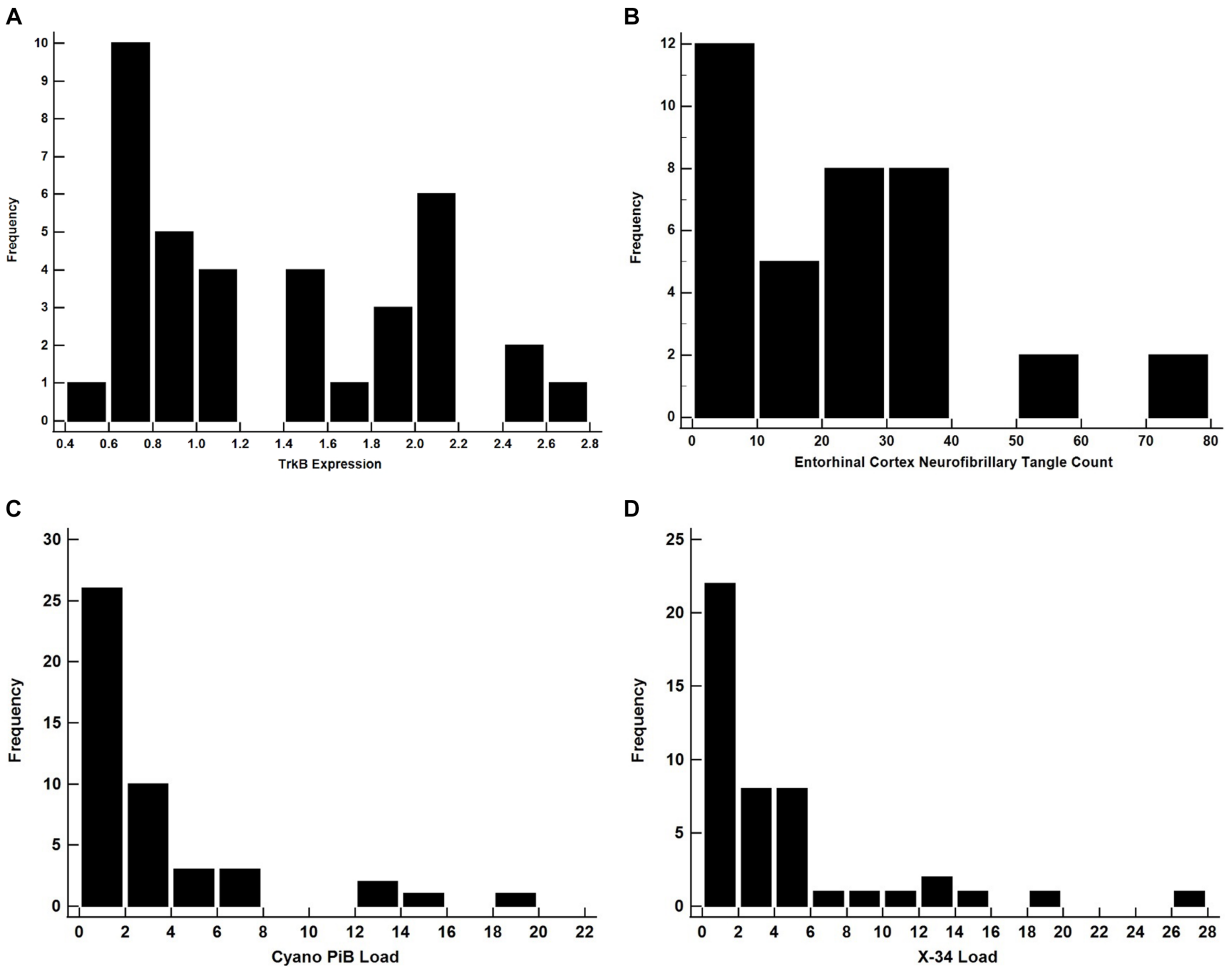
All of these distributions are likely to yield invalid results if used as a dependent variable in a linear regression model. Histograms for TrkB Expression **(A)**, Entorhinal Cortex NFT Count **(B)**, Cyano-PiB Load **(C)**, and X-34 Load **(D)**.

The analyses examining the association between TrkC expression and entorhinal cortex NFT load also showed that the results of the linear and robust regression models differed due to the dependent variable’s non-Gaussian distribution (Figures 3A,B). TrkC did not correlate with age (*r* = −0.18, *p* = 0.27) or education (*r* = −0.08, *p* = 0.65) and did not differ between males and females (*p* = 0.44) The linear model results indicated a statistically significant association between TrkC expression and entorhinal cortex NFT load (*β* = −15.18, SE = 6.50, *p* = 0.03, *R*^2^ = 0.46) while the results from robust model were not statistically significant (*β* = −12.13, SE = 6.10; *p* = 0.06, *R*^2^ = 0.32; Table 3). Here, the robust regression model yielded a smaller regression coefficient and accounted for far less variance in the dependent variable than the linear model. Although the SE values were comparable, it is important to note that the linear model’s value of p indicated a statistically significant association but not for the robust model. This also exemplifies how the results of a linear model are biased when the error distribution of the dependent variable does not meet the assumption of normality. Residual plots for the linear and robust regression models and indicate a lack of correlation between the fitted and residual values (Figures 2C,D). The permutation linear regression models yielded results that were identical to the regular linear regression model except for the *R^2^* value, which was nine percentage points lower.

It is important to note that the original analyses consisted primarily of non-parametric Spearman correlations intended to address specific hypotheses (Ginsberg et al., 2019). The analyses presented here do not contradict the findings of the original study but show how robust regression may be used as an alternative to linear regression when the error distribution of a dependent variable does not meet the assumption of normality.

### Dataset 2

Demographic, cognitive, and neuropathologic data for the NCI, MCI, and AD cases are shown in Table 1.

**Figure 4:**
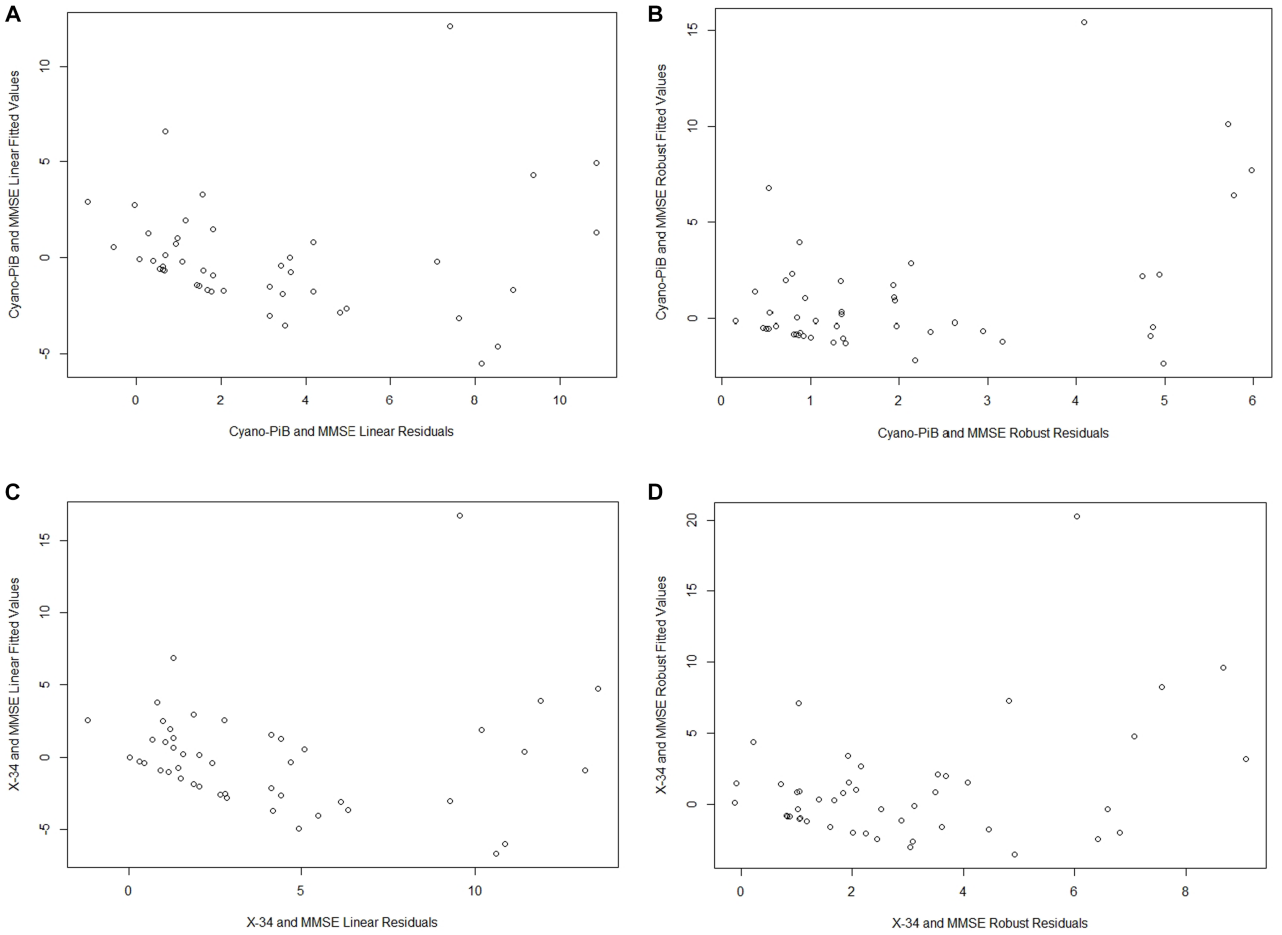
All of the plots indicate a lack of correlation between the fitted values and residuals from each model (**A** - Cyano-PiB and MMSE Linear Model; **B** - Cyano-PiB and MMSE Robust Model; **C** - X-34 and MMSE Linear Model; **D** - X-34 and MMSE Robust Model).

Both cyano-PiB and X-34 load in the precuneus cortex failed to meet the assumption of normality (*p* < 0.001 for both, Table 2) as their respective histograms indicated significant right-skewness (Figures 3C,D). Cyano-PiB did not correlate with age (*r* = 0.01, *p* = 0.97), but did show a weak correlation with education (*r* = −0.32, *p* = 0.03). Males and females did not differ on cyano-PiB load (*p* = 0.69). When the MMSE is used as a predictor of cyano-PiB load both the linear model (*β* = −0.32, SE = 0.06, *p* < 0.001, *R*^2^ = 0.54) and the robust model (*β* = −0.18, SE = 0.05, *p* < 0.001, *R*^2^ = 0.21) yielded a significant association (Table 3). Although both models revealed statistically significant associations, we found major differences in the regression coefficients and *R*^2^ values. Specifically, the values in the linear model were substantially larger than those in the robust model indicating that the skewed distribution of cyano-PiB values led to inflated estimates in the linear model. Residual plots for the linear and robust regression models indicated a lack of correlation between the fitted and residual values (Figures 4A,B). Although the *p*-values of both models indicate statistically significant associations, the strength of the associations in each model differed substantially with the robust model providing a more conservative estimate and accounted for less variance in the dependent variable. These differences highlight the need to de-emphasize interpretations that are based primarily on p-values. The permutation model yielded results identical to the regular linear regression model except that the *R*^2^ value was five percentage points lower (Table 4). The permutation models for cyano-PiB and X-34 both achieved their results in five permutations.

The analyses for MMSE and X-34 load also demonstrated that estimates for the strength of association can differ widely depending on whether a linear or robust regression model is performed. X-34 did not correlate with age (*r* = −0.02, *p* = 0.88), but showed a weak correlation with education (*r* = −0.33, *p* = 0.02). Males and females did not differ on cyano-PiB load (*p* = 0.82). Both the linear model for the MMSE and X-34 (*β* = −0.37, SE = 0.07, *p* < 0.001, *R*^2^ = 0.52) and the robust model (*β* = −0.24, SE = 0.05, *p* < 0.001, *R*^2^ = 0.15; Table 3) demonstrated a statistically significant association. However, there was a notable difference in the numeric value of the regression coefficients and a 3.5-fold difference in the R^2^ value. Residual plots for the linear and robust regression models indicate a lack of correlation between the fitted and residual values (Figures 4C,D). The permutation model yielded results identical to the regular linear regression model except for the *R*^2^ value, which was five percentage points lower (Table 4). Like the cyano-PiB example, the differences between the linear and robust models for the MMSE and X-34 association indicate the improtance of considering whether the assumption of normality for a dependent variable is met before using a linear regression model.

It should be noted that the analyses in Mi et al. (2023) used the MMSE as the dependent variable with cyano-PiB and X-34 as independent variables. Here, we used cyano-PiB and X-34 as the dependent variables since their distributions are more like those of other biological variables used in neuroscience studies.

## Discussion

Using two different independent datasets from published postmortem neurodegenerative disease studies (Ginsberg et al., 2019; Mi et al., 2023), we demonstrated how robust regression can be used as an alternative to linear regression when the error distribution of a dependent variable does not meet the assumption of normality. The examples used in the present study show how estimates for regression coefficients, SE, *p*-values, and *R*^2^ values can be significantly biased using linear regression models when the dependent variable does not meet the assumption of normality.

While it is known that parametric tests are robust to small deviations of the normality assumption (Schmider et al., 2010; Ghasemi and Zahediasl, 2012), this assertion assumes that there are no outliers and that the sample size is large. This is important as many translational neuroscience studies use smaller sample sizes. Although the examples evaluated had samples sizes of *n* = 37 and *n* = 46, which some may consider to be sufficiently large for a linear regression, the distributions of the dependent variables in these examples shows that their skewness precludes the use of linear regression. A more reliable estimate was obtained using a robust regression.

Justification for the use of parametric tests when the sample size is *n* ≥ 30 is based on the Central Limit Theorem (CLT), which states that a distribution of several different means tends to be normal, or approximately normal, when sample sizes approach *n* = 30 (Kwak and Kim, 2017). This conceptualization of the CLT is a fundamental concept taught in introductory statistics courses and has led to a fallacy that a sample size of at least *n* = 30 that parametric tests can be used without regard to a dependent variable’s error distribution. The present examples demonstrate that this “*n* = 30 guideline” should not be used to determine whether the use of parametric statistical tests is appropriate. Simulation studies have shown that at sufficiently large sample sizes (*n* = 100–1,000) parametric tests are robust to significant deviations from the normality assumption (Knief and Forstmeier, 2021). Since, in many areas of neuroscience obtaining sample sizes of these magnitudes is often impractical given that resources such as animals and human postmortem tissue are limited, many neuroscience studies are carried out with smaller sample sizes like those in current datasets (*n* = 37 and *n* = 46).

Another important aspect of the analyses presented here is that the robust regression models yielded more conservative estimates of associations and variance accounted for in the dependent variable. This observation was particularly striking in the regression models used for Dataset 2 where the robust regression coefficients *R*^2^ values were markedly lower than those in the linear models (Tables 3, 4). This example shows how the results of linear regression may provide artificially high estimates of associations when dependent variables do not meet the assumption of normality. The examples in Dataset 1 demonstrate an additional problem that arises when linear models are used when a dependent variable is significantly skewed. The value of ps from the linear models in Dataset 1 indicated statistically significant associations while the robust models yielded non-significant value of ps. In this example the regression coefficients did not differ substantially between the two models. However, differences in the models *p*-values would lead to very different conclusions about the statistical significance of the findings. In this regard, we acknowledge that the analyses for TrkC and entorhinal cortex NFT count may actually suggest the presence of a significant association given that both the linear and permutation models yielded p-values that were < 0.05 while the robust model’s p-value was 0.06. In the absence of a ground truth it is unclear whether the robust model represents the true estimate of the association and is a limitation of this study.

It is rare that dependent variables in translational neuroscience studies meet the assumption of normality (Sawada, 2021) and as a result robust regression can and should be more widely used in order to provide more accurate and reliable estimates, particularly when the sample size is small. A major impediment to the wider use of robust regression is that it is not typically included in graduate level statistics courses among neuroscience training programs. While robust regression methods may be encountered through statistics and methodology seminars as well as from statistically focused faculty, incorporating robust regression into required statistics classes in neuroscience graduate programs will go a long way toward the on-going efforts to promote and enhance rigor and reproducibility in the field. To help facilitate the use of robust regression, we have included the *R* code as Supplementary material (see Supplementary file). Readers will find that the syntax structure of the linear and robust models is similar with the main difference being the statistical function (lm versus lmRob) used in the analyses.

## Conclusion

It is rare that dependent variables in neuroscience meet the assumption of normality allowing for the use of linear regression models. Given the highly skewed tendencies of these variables and in some instances modest sample sizes, robust regression is a viable alternative that should be used more often. Given the level of attention being paid to rigor and reproducibility of neuroscience findings, incorporating robust regression as part of course curricula for graduate programs in neuroscience will go a long way toward increasing the statistical rigor of published neuroscience studies.

## Data availability statement

The data analyzed in this study is subject to the following licenses/restrictions: Demographic, cognitive, and neuropathological data is available upon request from the Rush Alzheimer’s Disease Research Center at: https://www.radc.rush.edu/requests.htm. Availability for neurotrophin receptor and X-34/cyano-PiB data is made through request to the collaborating authors (MM-A, SDG, and MDI). Requests to access these datasets should be directed to MM-A, michael.malekahmadi@bannerhealth.com.

## Ethics statement

The studies involving humans were approved by Rush University Institutional Review Board. The studies were conducted in accordance with the local legislation and institutional requirements. Written informed consent for participation in this study was provided by the participants’ legal guardians/next of kin.

## Author contributions

MM-A: Conceptualization, Formal analysis, Methodology, Writing – original draft, Writing – review & editing. SDG: Data curation, Writing – review & editing. MJA: Data curation, Writing – review & editing. SC: Writing – review & editing. MDI: Data curation, Writing – review & editing. EEA: Data curation, Writing – review & editing. SEP: Writing – review & editing. EJM: Funding acquisition, Project administration, Writing – review & editing.
