# Supplementary material for: Application of robust regression in translational neuroscience studies with non-Gaussian outcome data
Source: Front Aging Neurosci. 2024 Jan 24;15:1299451. doi: 10.3389/fnagi.2023.1299451 (PMC10847267; doi:10.3389/fnagi.2023.1299451)
Supplement: Supplementary file 1 [file Table_1.DOCX]

***R code for linear regression models***

*GCS and NTRK2*

gcs_ntfk2_linear<-lm(ntrk2tk~age+sex+educ+apoe+gcs, data = ca1_single_cell_example)

*NTRK3 and Entorhinal Cortex Neurofibrillary Tangle Load*

ntrk3_ec_nft_linear<-lm(ec_nft~age+sex+educ+apoe+ntrk3tk, data = ca1_single_cell_example)

*Cyano-PiB and MMSE*

cyano_pib_mmse_linear<-lm(cyano_pib_load_perc_area~age_death+sex+educ+mmse, data = vglut_data)

*X-34 and MMSE*

x34_mmse_linear<-lm(x_34_load_perc_area~age_death+sex+educ+mmse, data = vglut_data)

***R code for robust regression models***

install(robust)

install(robustbase)

library(robust)

library(robustbase)

*GCS and NTRK2*

gcs_ntfk2_robust<-lmRob(ntrk2tk~age+sex+educ+apoe+gcs, data = ca1_single_cell_example)

*NTRK3 and Entorhinal Cortex Neurofibrillary Tangle Load*

ntrk3_ec_nft_robust<-lmRob(ec_nft~age+sex+educ+apoe+ntrk3tk, data = ca1_single_cell_example)

*Cyano-PiB and MMSE*

cyano_pib_mmse_robust<-lmRob(cyano_pib_load_perc_area~age_death+sex+educ+mmse, data = vglut_data)

*X-34 and MMSE*

x34_mmse_robust<-lmRob(x_34_load_perc_area~age_death+sex+educ+mmse, data = vglut_data)

***R code for linear permutation regression models***

*GCS and NTRK2*

gcs_ntfk2_perm<-lmp(ntrk2tk~age+sex+educ+apoe+gcs, perm=”Exact”, data = ca1_single_cell_example)

*NTRK3 and Entorhinal Cortex Neurofibrillary Tangle Load*

ntrk3_ec_nft_perm<-lmp(ec_nft~age+sex+educ+apoe+ntrk3tk, perm=”Exact”, data = ca1_single_cell_example)

*Cyano-PiB and MMSE*

cyano_pib_mmse_lperm<-lmp(cyano_pib_load_perc_area~age_death+sex+educ+mmse, perm=”Exact”, data = vglut_data)

*X-34 and MMSE*

x34_mmse_perm<-lmp(x_34_load_perc_area~age_death+sex+educ+mmse, perm=”Exact”, data = vglut_data)
